# Processing emotional prosody in a foreign language: the case of German and Hebrew

**DOI:** 10.1007/s41809-022-00107-x

**Published:** 2022-08-18

**Authors:** Vered Shakuf, Boaz Ben-David, Thomas G. G. Wegner, Patricia B. C. Wesseling, Maya Mentzel, Sabrina Defren, Shanley E. M. Allen, Thomas Lachmann

**Affiliations:** 1 Communication, Aging and Neuropsychology Lab (CAN Lab), Baruch Ivcher School of Psychology, Reichman University (IDC), Herzliya, Israel; 2 Department of Communications Disorders, Achva Academic College, Arugot, Israel; 3 Department of Speech-Language Pathology, University of Toronto, Toronto, ON Canada; 4 KITE, Toronto Rehabilitation Institute, University Health Networks (UHN), Toronto, ON Canada; 5 Cognitive and Developmental Psychology Unit, Center for Cognitive Science, University of Kaiserslautern, Kaiserslautern, Germany; 6 Chemnitz University of Technology, Chemnitz, Germany; 7 Psycholinguistics and Language Development Unit, Center for Cognitive Science, University of Kaiserslautern, Kaiserslautern, Germany; 8 Centro de Investigación Nebrija en Cognición (CINC), Universidad Nebrija, Madrid, Spain; 9 University of Leuven, Leuven, Belgium

**Keywords:** Cross-cultural, Emotional prosody, Semantics, Language comparison, Universality

## Abstract

This study investigated the universality of emotional prosody in perception of discrete emotions when semantics is not available. In two experiments the perception of emotional prosody in Hebrew and German by listeners who speak one of the languages but not the other was investigated. Having a parallel tool in both languages allowed to conduct controlled comparisons. In Experiment 1, 39 native German speakers with no knowledge of Hebrew and 80 native Israeli speakers rated Hebrew sentences spoken with four different emotional prosodies (anger, fear, happiness, sadness) or neutral. The Hebrew version of the Test for Rating of Emotions in Speech (T-RES) was used for this purpose. Ratings indicated participants’ agreement on how much the sentence conveyed each of four discrete emotions (anger, fear, happiness and sadness). In Experient 2, 30 native speakers of German, and 24 Israeli native speakers of Hebrew who had no knowledge of German rated sentences of the German version of the T-RES. Based only on the prosody, German-speaking participants were able to accurately identify the emotions in the Hebrew sentences and Hebrew-speaking participants were able to identify the emotions in the German sentences. In both experiments ratings between the groups were similar. These findings show that individuals are able to identify emotions in a foreign language even if they do not have access to semantics. This ability goes beyond identification of target emotion; similarities between languages exist even for “wrong” perception. This adds to accumulating evidence in the literature on the universality of emotional prosody.

**Supplementary Information:**

The online version contains supplementary material available at 10.1007/s41809-022-00107-x.

## Introduction

Perceiving and comprehending emotions in speech is crucial in almost every type of conversation, both personal and professional. Successful interactions depend on being able to correctly identify the emotions conveyed by others when talking with them; not being able to do so can lead to negative social consequences including reduced relationship well-being and depression (Carton et al., 1999; Phillips et al., 2003). However, understanding emotions in spoken language is a complex process involving many linguistic and non-linguistic factors. Much research has been devoted to the processing of non-linguistic cues in emotional speech, such as facial expression (Elfenbein & Ambady, 2002; Radice-Neumann et al., 2007). In the past two decades, an increasing amount of research has also been devoted to linguistic factors active in emotional speech, such as semantics and prosody (Ben-David et al., 2016; Dricu & Frühholz, 2020; Gruber et al., 2020; Marc D. Pell et al., 2009; Zupan et al., 2009). Semantics refers to the meaning of words in the sentence, as well as the meaning of the sentence as a whole (Löbner, 2013; Rom et al., 2005). Prosody (tone of speech) is the speech information which cannot be reduced to the individual segments (consonants and vowels) or their juxtaposition—primarily the suprasegmental features of speech including rhythm, stress, and intonation (Mixdorff & Amir, 2002; Shriberg et al., 2000; van de Velde et al., 2019). In what follows, we limit our use of the term semantics to refer to the meaning of individual sentences, and the term prosody to refer to emotional prosody.

Semantics and prosody are separate but not separable channels in the processing of spoken language (Ben-David et al., 2016). For instance, Ben David and colleagues showed that when native speakers of English are asked to determine whether spoken sentences in their language convey particular emotions (anger, fear, sadness, happiness), their perception is influenced by both channels, even if one of the factors—either semantics or prosody—is irrelevant for the task. Notably, listeners tend to give more weight to the prosodic channel than to the semantics (Ben-David et al., 2016), indicating the important role that prosody plays in the perception of emotional speech. In addition, prosody facilitates decoding the semantic content of spoken language since it can be used for both speech recognition (i.e. increasing speed and accuracy) and linguistic processing (i.e. supporting computation of meaning; Lieske et al, 1997; disambiguating syntactic information; Snedeker & Trueswell, 2003).

In conditions where semantic and prosodic information are both fully available, such as interactions in one’s native language in ideal listening conditions (quiet background, conducted singly, see, e.g., Hadar et al., 2016; Nitsan et al., 2019), one has the advantage of being able to use both channels to produce and understand emotions. The literature also shows that listeners can correctly identify the emotional intention of the speaker based on the prosody alone (e.g., Ben-David et al., 2016; Jacob et al, 2014; Roche et al., 2015; Scherer et al., 2001). Emotional meanings in the prosody are conveyed by accompanying changes in several acoustic parameters of speech. Among these are mean vocal pitch, pitch range (or variation), and speech rate, all of which appear to differentiate well among discrete emotional categories (Banse & Scherer, 1996; Mozziconacci, 1995; Pannese et al., 2016; Pell, 2001; Williams & Stevens, 1972, for review see An et al., 2017; Batliner et al., 2011; Cowen et al., 2019; Pannese et al., 2016; Zupan et al., 2009).

Identifying emotions primarily on the basis of prosody is relevant in several social situations. Prosody can be the main means to convey emotions when visual cues are missing (e.g. in a phone call) or when the semantics is distorted (e.g. accented speech; Van Engen & Peelle, 2014). Individuals who have difficulty extracting information about emotions from facial expressions or body posture of an interlocutor can compensate using prosodic information (e.g. autism, traumatic brain injury; Ben-David et al., 2011b; Cicero et al., 1999; Icht et al., 2021, 2022; Zupan et al., 2009). Further, in addition to being a robust means of conveying emotions in speech within a specific language, prosody also appears to have several universal features (Pell et al., 2009, for review see Juslin & Laukka, 2003). For example, across cultures, sadness tends to be produced with a relatively low pitch/fundamental frequency (*f*_*0*_) and slow speaking rate, whereas anger, fear, and happiness tend to be produced with a moderate or high mean *f*_*0*_ and fast speaking rate. Anger and happiness usually display high *f*_*0*_ variation, whereas fear and sadness often exhibit less *f*_*0*_ variation (An et al., 2017; Scherer, 2003; Ueyama & Li, 2020). Although there are exceptions to these patterns, several prosodic attributes are typically reliable enough to serve as universal cues to recognize and distinguish emotions in speech without having semantic information.

This universality of prosody can at least partially be attributed to evolutionary reasons. Scholars as early as Darwin (1872) have claimed that the capacity to employ vocal cues for communication appears early in development of the species and the individual. Indeed, even neonates and non-humans have been found to be sensitive to emotional prosody. Brain imaging studies have shown that the cerebral specialization for emotional processing develops in the first days of life, such that babies can distinguish between different emotional vocal cues (Cheng et al., 2012). Further, children as young as 6 months of age have been shown preferred positive emotional Adult Directed Speech (ADS) over Infant Directed Speech (IDS), and positive over negative affective speech (Singh et al., 2002). Researchers have attributed this to the importance for survival of decoding prosody, in that positive (e.g., happy) prosody is more likely linked to nonthreatening and care-giving conspecifics. This has led researchers to suggest that humans as well as animals are biologically pre-programmed to identify prosodic features (Lahvis et al., 2011).

Numerous studies reveal that individuals are able to use these universal features of prosody to reliably identify emotions at above-chance levels across languages, including in languages that they do not know (for a recent review and meta-analysis, see Laukka & Elfenbein, 2021). Three approaches have typically been used. In one set of studies, native speakers of several languages are asked to identify emotions in utterances of one particular language. For instance, Van Bezooijen, Otto and Heenan (1983) asked native speakers of Dutch, Taiwanese, and Japanese to identify disgust, surprise, shame, interest, joy, fear, contempt, sadness, and anger in one phrase (*twee maanden zwanger* ‘two months pregnant’) spoken in Dutch and found above chance accuracy for all groups. Similar results were obtained by Scherer and colleagues (2001) using 30 semantically-anomalous pseudo-sentences, spoken with emotional prosodies by German-speaking actors. In the second set of studies, native speakers of one language are asked to identify emotions in utterances of several languages. For example, Thompson and Balkwill (2006) asked native speakers of English to identify joy, anger, fear, and sadness in two semantically neutral sentences (*The* *bottle* *is* *on* *the* *table*, *The* *leaves* *are* *changing* *color*) spoken with emotional prosody in each of five languages: English, German, Japanese, Chinese, and Tagalog, with above chance identification. Again, similar results were obtained with pseudo-sentences (Pell et al., 2009). Finally, a few studies combine these two methods in a balanced design, in which native speakers of two languages are asked to identify emotions in utterances from each of those languages. For example, McCluskey and Albas (1981) asked native speakers of English and Mexican Spanish to identify anger, love, happiness, and sadness in digitally manipulated utterances (low-pass-filtered utterances with the content chosen by the speaker) in each of the two languages. A similar method was used by Paulmann and Uskul (2014), with English and Chinese native speakers listening to pseudo-sentences in English and Chinese (e.g., for English: *Flotch deraded the downdary snat*). In all of these studies, participants identified all emotions in all languages consistently with accuracy above chance level. These results suggest that at least some aspects of prosody must be universal.

These studies highlighted the universality of prosodic cues in identifying emotions in speech. However, no information was obtained on the options rejected by the listener. For example, even though listeners choose an ‘anger’ response for a given utterance, they may also hear aspects of sadness. Such additional information is essential to understand the full complexity of the perception of prosodic cues in emotional speech. An alternative method, such as a rating scale, would make this subtle information more visible—for instance, if in the same case, the listener rated ‘anger’ as 6/6 and sadness as 4/6 on a six-point Likert scale. Lima and colleagues (2016) used a similar method to test identification of emotional prosody in participants with a developmental music disorder (as compared to participants with typical development). In their study participants judged three types of emotional stimuli: nonverbal vocalizations, facial expressions, and sentences containing neutral semantics but emotional prosody. Each stimulus was rated on 7-point scale indicating how much one of seven emotions (amusement, anger, disgust, fear, pleasure, relief, and sadness) was expressed. However, they analyzed the ratings for the non-intended emotions as a whole and did not look for systematic differences between them. Ben-David and colleagues (2016) also used a rating scale in their Test for Rating of Emotions in Speech (T-RES). In the T-RES, participants are asked to rate sentences spoken with emotional prosody four times, once for each of four discrete emotions (i.e., anger, fear, happiness, sadness). This tool has also been used to compare different groups of participants e.g., older vs. younger adults, participants with high-functioning autism spectrum disorders vs. participants with typical development, participants with tinnitus vs. participants with normal hearing; (Ben-David et al., 2019, 2020, 2016; Dor et al, 2022a, 2022b; Leshem et al., 2020, 2022; Oron et al., 2020; Taitelbaum-Swead et al., 2022). In the current study, we employed the T-RES to compare emotional prosody ratings across speakers of different languages. Further, we more fully exploited the advantages of this method by analyzing the non-intended emotions separately. This will allow us to reveal the nuances of similarities and differences across languages, specifically German and Hebrew.

Another common finding in this literature is a native-language advantage: emotions in spoken prosody are better identified in the listener’s native language than in a foreign language (Paulmann & Uskul, 2014; Pell et al., 2009; Scherer et al., 2001; Thompson & Balkwill, 2006). However, there is disagreement on whether the magnitude of this advantage is influenced by the degree of linguistic and cultural similarity between the native and foreign languages. Some studies have shown that the accuracy in identification of emotion improves as the cross-linguistic similarity becomes higher (Elfenbein & Ambady, 2002; Scherer et al., 2001). Other studies did not find this relation between language similarity and emotion identification accuracy. For example, native speakers of Spanish were no better at using prosodic cues to identify emotion in English than in Arabic (with higher versus lower similarity to Spanish; Pell et al., 2009; Thompson & Balkwill, 2006). In the current study, we compare Israeli native speakers of Hebrew with German native speakers of German. We test whether, even in languages that are highly disparate in structure and culture, we can still find similarities in the ratings of emotions presented by prosody.

Another factor that can influence the perception of emotional prosody in a foreign language is attitudes toward the culture and the language, in that knowing which language one hears may activate stereotypes since social groups and cultures are perceived differently (Cuddy et al., 2009). For example, German participants may activate stereotypes towards Israelis when they know that they are going to hear sentences spoken in Hebrew. However, research in this area suggests that these stereotypes should not influence individuals’ perceptions of or attitudes towards the language, because nationality attitudes and language attitudes are distinct and do not necessarily influence each other (Lehnert & Hörstermann, 2019). Therefore, we do not expect that attitudes towards a given culture would influence perceptions of emotional prosody on the related prosody. Nonetheless, we controlled for knowledge of the language to assess any potential effect of attitudes towards language and culture.

In the current study, we compared emotional prosody ratings of Israeli native speakers of Hebrew (with no knowledge of German) and German native speakers of German (with no knowledge of Hebrew). In Experiment 1, we compared ratings of the two groups on the Hebrew version of the T-RES, and in Experiment 2 we compared their ratings on the German version of the T-RES. It is particularly interesting to investigate German and Hebrew because they represent different language families and linguistic typologies; German is a Germanic language and Hebrew is a Semitic language. The phoneme inventory also differs across the two languages: while German has 21 consonants and 16 vowels, Hebrew has as many as 27 consonants and ten vowels, or five vowels in modern spoken Hebrew (see Hurley, 1992; for an overview; Wiese, 1996). In addition, there are very few cognates (words that sound and mean the same) across the two languages because they are from different language families, so it is unlikely that the participants will be able to use their lexical knowledge to deduce any semantic information from the other language. However, Mixdorff & Amir (2002) suggested that there are strong prosodic similarities across German and Hebrew in some respects. For example, the place of focus is marked in both languages by high accent command amplitudes and reduction of post-focal accents, whereas pre-focal accents remain almost unaffected (Mixdorff & Amir, 2002).

To our knowledge, only one previous study has directly compared emotion perception in this language pair (Pfitzinger et al, 2011), focusing on activation, valence, and dominance using the Self-Assessment Manikin (see e.g. Bradley & Lang, 1994). In one portion of the study, German and Hebrew speakers were asked to rate Hebrew uncontrolled utterances recorded in psychotherapy sessions (not balanced for length, emotional semantic or prosodic content, or amplitude). They found asymmetrical cross-language differences in perception. For example, Hebrew utterances judged as negative by native-Hebrew listeners were judged as positive by German listeners and vice versa. Unlike valence, the dominance and activation scales showed ratings that were more equivalent.

## The current study

The goal of the current study was to conduct a controlled investigation of the nuances of the universality of emotional prosody in the perception of discrete emotions across languages. To this end, in Experiment 1 we asked native speakers of German who did not speak or understand Hebrew to rate four discrete prosodic emotions (anger, fear, sadness, happiness) using the Hebrew version of the Test for Rating of Emotions in Speech (T-RES; Ben-David et al., 2019, 2016). We compared these results with those of native speakers of Hebrew using the same test. In Experiment 2, native speakers of Hebrew who did not speak or understand German rated the same emotions on the German version of the T-RES (Carl et al., 2022; Defren et al., 2018). We compared these results with those of native speakers of German using the same test.

The main advantage of the T-RES for cross-linguistic studies is that it has been adapted and validated in different languages. Since the tool is parallel across the versions, this allows us to have more controlled comparisons between languages than has been the case in previous studies (see Elfenbein & Ambady, 2002; Radice-Neumann et al., 2007 and Defren et al., 2018; Carl et al., 2022 for a comparison between the different versions). Another advantage of the T-RES is that it allows us to obtain a more detailed view of the full subjective perception of emotional prosody than is possible with a simple classification of emotions. By using a rating scale, we can gauge and compare the extent of the perception of the different emotions across languages and speaker groups. These two features of the T-RES combined together will allow us to test whether the perception of emotional prosody in an unknown language functions in the same way in both directions: German speakers to Hebrew and Hebrew speakers to German. Other important methodological features of the current study and the T-RES that enable us to go beyond the findings of previous related studies are discussed in the Method section.

The present study asks the following two research questions:

- 1) Are listeners able to identify emotions in prosody using a rating scale when access to lexical semantics is not available (i.e., when they do not know the language)? Specifically, can native speakers of German identify emotional prosody conveyed in Hebrew natural speech, and can Hebrew native speakers identify emotional prosody conveyed in German natural speech? Consistent with existing findings in the literature, we predict that this will be the case. If so, our findings will extend the existing evidence to a new language pair. We also predict that emotions considered (rated as second or third option) will also be similar across the two groups.
- 2) More importantly, what are the similarities and differences in identifying emotional prosody in an unknown language vs. in a native language? As a result of the subtlety of the rating scale used in the T-RES, we expect to uncover nuances and possible asymmetries in perception patterns between the languages and groups of speakers. In particular, we go beyond the ‘final response’ and test whether German native speakers rate the non-intended, non-chosen emotions the same in Hebrew as Hebrew native speakers do and vice versa.

## Design and procedure

The study was approved by the Ethics Committees of the University of Kaiserslautern and the Interdisciplinary Center (IDC) Herzliya. As the first step in the study, all participants received a short explanation regarding the experimental task, and signed an informed consent form. They then completed three self-report questionnaires to confirm inclusion criteria. Participants who met the criteria then performed the T-RES individually, in a sound-attenuated booth at their respective university lab. The Hebrew-speaking participants of Experiment 1 performed all three rating tasks while the Hebrew-speaking participants in Experiment 2 as well as the German-speaking participants in both Experiments only performed the Prosody-rating task. Stimuli were presented in four separate emotion-rating blocks: anger-rating, fear-rating, happiness-rating, and sadness-rating. Each trial began with the presentation of the audio file via WH-102 headphones, using a sampling rate of 22.05 kHz. This was followed by presentation of the instructions and the rating scale on the monitor. For each spoken sentence, the participants were asked to rate how much they agreed that the speaker conveyed a predefined emotion, using a 6-point Likert scale. For example, “How much do you agree that the speaker is conveying happiness? From 1—strongly disagree to 6—strongly agree.” Each rating block commenced with two practice trials. As the T-RES gauges the listener’s subjective perception of emotions, no feedback was provided in either the practice or experimental trials (i.e., there are no “right” or “wrong” answers).

Each of the 24 sentences was presented once in each of the four rating blocks (anger, fear, happiness, sadness), yielding a total of 96 trials per participant. To control for order effects, the order of the four emotion-rating blocks was counterbalanced (using a Latin square) and the order of the trials in each block was fully randomized (closely following the original T-RES study, see Table 2 in Ben-David et al., 2016).

In Experiment 1, a random half of the German participants were informed that the language they were about to hear was Hebrew (“informed” group). The other half, the “uninformed” group, received no information about the language. To ensure that none of the German “uninformed” group recognized the language of the spoken sentences, participants in this condition were asked upon completing the T-RES if the language was a real or artificial one and, if the former, which language it was. None of the participants recognized the language as Hebrew.

## Experiment 1

### Methods

#### Participants

Two groups of participants were recruited: Germans (*N* = 39; 18 females) and Israelis (*N* = 80; 58 females; as gender distribution was not equated across groups, *χ*^*2*^(1) = 4.09, *p* = 0.043, we used gender as a between-group factor in all analyses). To control for possible effects of pre-existing attitudes related to Hebrew and to Israel, German participants were randomly assigned to two groups: one half of the participants was told they were going to hear Hebrew sentences (*informed*, *N* = 20) and the other half received no information about the language (*uninformed*, *N* = 19). The two subgroups did not differ in age, *t*(37) = 0.97, *p* = 0.34.

*Inclusion criteria:* Both groups of participants adhered to the following criteria. First, all participants ranged in age from 18 to 30 years (Germans: *M* = 24 years, *SD* = 3.1; Israelis: *M* = 23.6 years, *SD* = 2.2; *t*(117) = .96, *p* = 0.34). Second, all participants were students at an academic institute in their respective country (Germans: University of Kaiserslautern; Israelis: The Interdisciplinary Center [IDC], Herzliya), and received either course credit or monetary compensation (€10 or 30 NIS) for their participation. Third, all participants were native speakers of their respective language—either German or Hebrew—as assessed by a self-report questionnaire. For the German participants we also ensured that none of the participants had knowledge of Hebrew (in a questionnaire administered before the study). Fourth, all participants were in good health and had no history of speech, language, or hearing problems, as assessed by a self-report questionnaire. Finally, as depression has been found to affect perception of emotions (Carballedo et al., 2011) we made sure that none of our participants showed depression symptoms, as assessed by the Depression, Anxiety and Stress Scale (DASS21; Lovibond et al., 1995), administered in the respective language.

#### Materials

##### Test of rating of emotions in speech (T-RES)

**Fig. 1:**
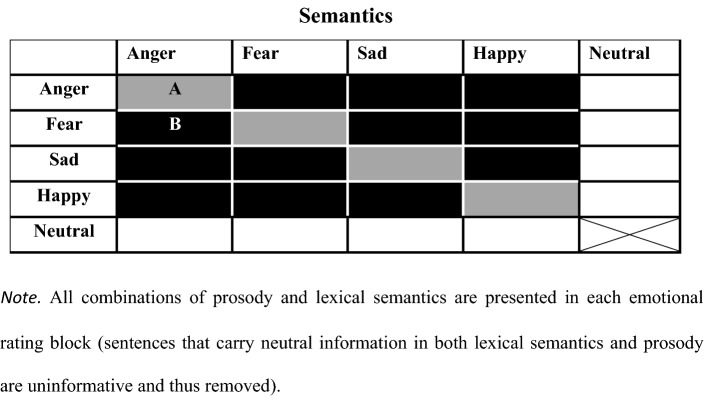
General design of the T-RES (Test of Rating of Emotions in Speech)

The study was conducted using the Hebrew version of the T-RES (Ben-David et al., 2019; Oron et al., 2020; Shakuf et al., 2016). The T-RES has been proven to be a useful tool for testing the perception of emotions in speech. It was first developed and validated in English (for details on the creation of the tool and validation process see Ben-David et al., 2013, 2016, 2011a, b). It was further adapted and validated to Hebrew (Ben-David et al., 2016) and recently to German (Defren et al., 2018), and has been used to test and compare several different groups, e.g., younger adults with normal hearing (Ben-David et al., 2016), older adults (Ben-David et al., 2019), students with high functioning Autism Spectrum Disorder (Ben-David et al., 2019, 2020), individuals with forensic schizophrenia (Leshem et al., 2020) and adults with tinnitus (Oron et al., 2020). The T-RES is comprised of sentences expressing particular emotions in the semantic and prosodic channels, which participants rate as to the degree of the emotion conveyed. Four emotional categories are used: anger, fear, happiness, and sadness. These emotions were chosen as they are expressed universally (Zupan et al., 2009), as well as being easily recognized and distinguished in prosody (Juslin & Laukka, 2003; Scherer et al., 2001). The test also includes a neutral category as a baseline condition for performance. Sentences conveying each of the five semantic categories were recorded using the five different prosodies. The combination of neutral prosody and neutral semantic content was deemed uninformative (see Ben-David et al., 2016) and removed. The final experimental set comprised 24 sentences, in which each semantic category was represented once in each of the tested prosodies, generating a 5 (lexical semantics) X 5 (prosody) matrix (minus the one removed cell), as shown in Fig 1. For example, the left column of Fig 1 presents sentences with angry semantic content, spoken with a different prosody in each cell. This means that the lexical semantics and prosody for a given sentence can either match (e.g., cell A in Fig 1) or mismatch (e.g., cell B in Fig 1). All sentences were rated as distinctive exemplars of their respective prosodic categories by a group of trained raters (following the procedures discussed in Ben-David et al., 2011a, b, 2013). Digital audio files were equated with respect to their root-mean-square amplitude. Sentence duration was equated across emotional prosodic and semantic categories. For full description of the T-RES, see Ben-David and colleagues (2016, 2019). An on-line version of the English, Hebrew and German versions can be found at https://www.canlab.idc.ac.il/

One main advantage of the T-RES is its use of natural sentences that carry semantic content, in contrast with most previous studies that have used pseudo-sentences or words devoid of semantic content (e.g., Pell et al., 2009). Using natural sentences means that the difference between the native language of the listeners and the language in which the utterances are produced can be precisely defined, whereas pseudo-sentences may not accurately reflect the linguistic characteristics of the language that they simulate. In the few previous studies that did use natural speech, only one or two different utterances were used as stimuli (but see Rochman et al., 2008). This results in unequal combinations of emotions conveyed along the channels of prosody and lexical semantics, which in turn can create a bias in performance (Melara & Algom, 2003). Further, it is unclear whether the performance of the participants would generalize beyond the limited situation of only one or two utterances.

Another defining characteristic of the T-RES is its use of carefully controlled stimuli, in contrast with other studies (e.g. Bowers et al., 1991; Pfitzinger et al., 2011) that have used recordings of daily speech (i.e., natural emotional scenes). For all versions of the T-RES, the stimuli were recorded by a professional actress who was a native speaker of the relevant language. Despite the artificial nature of this condition, it provides better control over the variability in the acoustics of the recorded material. More importantly for cross-linguistic studies, it yields a more intense and prototypical expression of the specific emotion (cf. Mitchell, 2006; Ben-David et al., 2016). In addition, the sentences are equated on linguistic characteristics across emotional categories including word frequency, length and number of phonemes (Ben-David et al., 2019). In sum, the T-RES presents listeners with a set of sentences with equal combinations of emotions across prosody and lexical semantics, and a sample size more likely to yield generalizable results.

For Experiment 1 we used the 24 sentences from the Hebrew version of the T-RES (Ben-David et al., 2019). See Supporting Information S1 for the full list of Hebrew sentences.

For Experiment 2, we used the 24 sentences from the German version of the T-RES (Defren et al., 2018). See Supporting Information S2 for the full list of German sentences.

## Results

### Germans identification of Hebrew emotional prosody

The first analysis focused only on the German participants, whose mean ratings on the T-RES are shown on the right half of Table 1. We tested whether the German participants could identify the emotions conveyed in the Hebrew sentences in the T-RES based on prosodic cues. We used an ANOVA to test the difference between the average ratings of sentences that present the rated emotion in the prosody versus sentences that did not (emotion identification). For example, the average prosodic ratings for anger in Hebrew sentences spoken with angry prosody should be very high, as the prosody conveys the rated emotion (rated emotion present). In contrast, the average prosodic ratings for anger in Hebrew sentences spoken with non-angry prosody (fear, happiness, sadness) should be very low, as the prosody does not convey the rated emotion (rated emotion absent). The difference between these two averages represents the extent of identification of prosodic emotions.

**Table 1:** Average ratings on a 6-point Likert scale for the Israeli and German groups with mean emotional ratings of prosodic categories in the T-RES Hebrew sentences

Israeli group (*N* = 80)					German group (*N* = 39)				
Rating scale					Rating scale				
Prosody	Anger	Fear	Sad	Happy	Prosody	Anger	Fear	Sad	Happy
Anger	5.9	1.5	1.5	1.1	Anger	5.7	1.6	1.4	1.3
Fear	2.8	4.8	2.3	2.0	Fear	2.9	4.4	2.7	2.3
Sad	1.4	3.1	5.8	1.1	Sad	1.3	3.8	5.6	1.3
Happy	1.2	1.1	1.2	5.4	Happy	1.9	2.3	2.5	4.7

We conducted a mixed model ANOVA with rated emotion (4: anger, fear, happiness, sadness) and prosodic emotion identification (2: Rated emotion present vs. absent) as within-participants variables, and language information (2: informed vs. uninformed), block order (4) and gender (2) as between-participants variables. The analysis showed a large prosodic emotion identification effect, *F*(1,33) = 12.82, *p* < 0.001, *η*_*p*_^*2*^ = 0.98, but language information had no significant main effect, *F*(1,33) = 1.38, *p* = 0.25, nor a significant interaction with prosodic identification, *F*(1,34) = 0.08, *p* = 0.79. Because none of the German participants understood Hebrew, we expected that the Hebrew semantic content would have no significant effect on their ratings. Nonetheless, we tested whether the semantic content of the sentences had an effect on prosodic ratings using a mixed model repeated measures ANOVA with Hebrew semantics (2: neutral vs. emotional) as a within-participants variable and language information (2: informed vs. uninformed) as a between-participants variable. This allowed us to compare baseline sentences that carried neutral Hebrew semantic content with sentences that carried emotional Hebrew semantic content. As expected, results indicated that the prosodic ratings of sentences with neutral and emotional semantics did not differ, *F*(1,33) = 1.09, *p* = 0.30, and that this factor did not interact significantly with language information *F*(1,33) = 0.11, *p* = 0.74. These results support our assumption that the German participants had no access to the semantic content of the sentences and hence their ratings were based only on the prosody. Therefore, we averaged across lexical semantics in the following analyses. Furthermore, results suggest that information about the language did not have an effect on the ratings of the German participants. As a result, we merged the ‘informed’ and ‘uninformed’ participants into one group for all subsequent analyses.

### Comparison of Hebrew native speakers and German native speakers

**Fig. 2:**
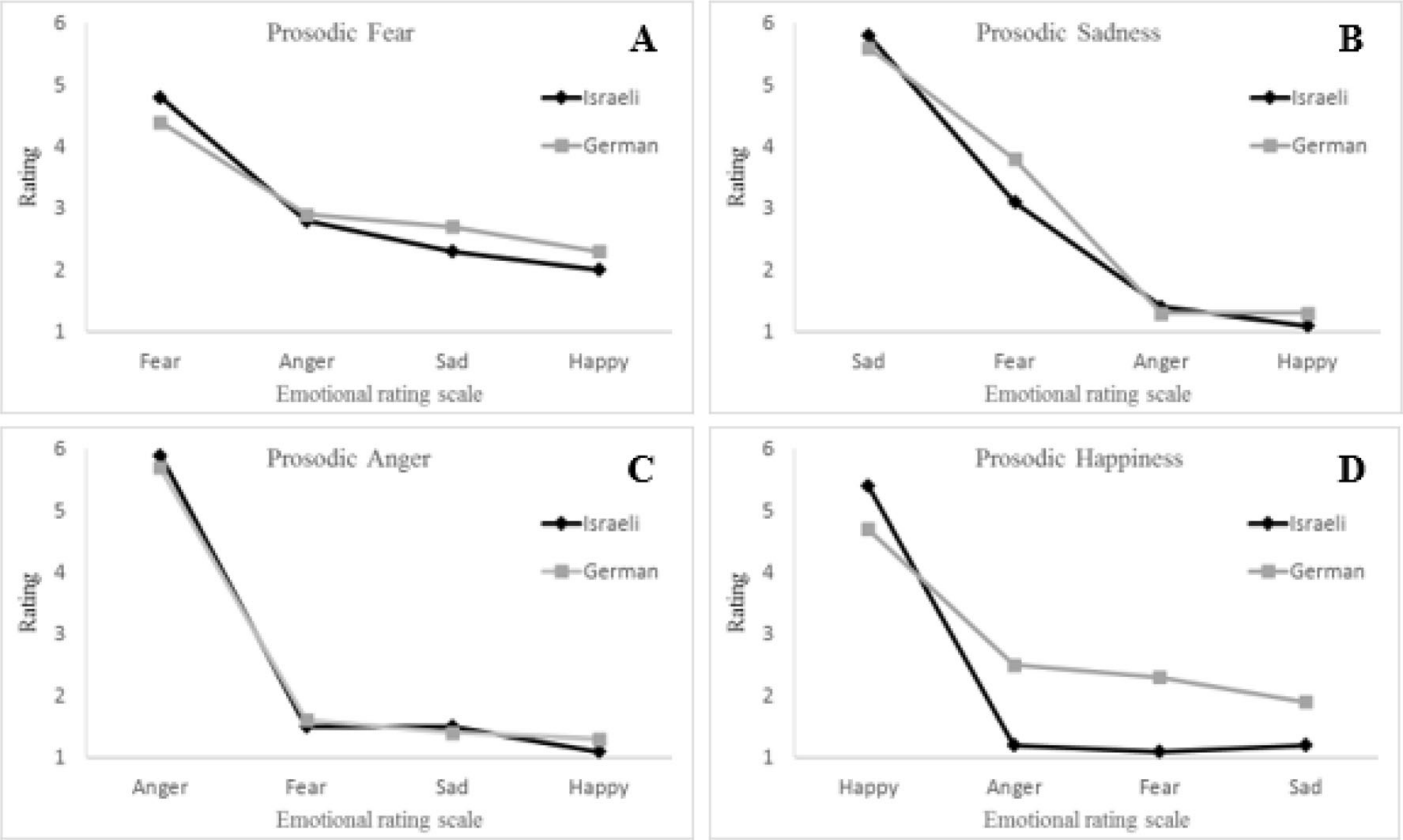
Average ratings of prosodic emotions in the T-RES Hebrew sentences. Emotional ratings scale for fear, sadness, anger and happiness

In the second analysis we compared the ratings of the German native speakers (who spoke no Hebrew) with the ratings of Hebrew native speakers, for the same sentences taken from the Hebrew T-RES. Table 1 and Figure 2 present the mean ratings on the four emotional rating scales of the respective emotional prosodic categories in the T-RES, for the two groups. We conducted a mixed model repeated measures ANOVA with group membership (2: Germans vs. Israelis), block order (4) and gender (2) as between-participants variables, and with rated emotion (4: anger, happiness, fear, sadness) and prosodic emotion identification (2: Rated emotion present vs. absent) as within-participants variables. First, we found a large main effect for prosodic emotions identification. *F*(1, 103) = 31.52, *p* < 0.001, *η*_*p*_^*2*^ = 0.97, that significantly interacted with group membership, *F*(1, 103) = 43.1, *p* < 0.001, *η*_*p*_^*2*^ = 0.3. Follow-up separate analyses revealed that both groups were highly accurate in identifying the prosody, *F*(1,75) = 27.92, *p* < 0.001, *η*_*p*_^*2*^ = 0.97, and *F*(1,34) = 12.20, *p* < 0.001, *η*_*p*_^*2*^ = 0.98, for Israelis and Germans, respectively. However, the extent was slightly larger for the Israeli group than for the German group (3.8/5 vs 3/5 on scale ranging from 0 to 5). This difference is consistent with expectations based on findings of a native-language advantage (Paulmann & Uskul, 2014; Pell et al., 2009; Scherer et al., 2001; Thompson & Balkwill, 2006).

### Identification of discrete emotions

As the last step of analysis, we looked at the average ratings of each prosodic emotion separately. Figure 2 highlights the many similarities in nuances of the ratings; two main features can be derived. First, for both groups, on all four prosodies, the highest scores were given on the respective target-emotion rating. For example, sentences spoken with angry prosody were rated higher on anger than on any other emotional rating (fear, happiness, sadness). Second and more interesting, for both groups, the rating trends on the non-respective emotions were similar as well.

To examine the first feature, we conducted a set of t-tests, separately for each group, comparing ratings of a prosodic emotion rated on its respective scale with ratings of the same prosody rated on all other scales. For example, for sentences spoken with angry prosody, we examined whether the highest ratings were given for anger versus sadness, happiness and fear. This was confirmed for all four prosodies, for Germans, *t*(38) > 3.4, *p* < 0.001, and for Israelis *t*(79) > 8.7, *p* < 0.001.

To examine the second feature, we conducted separate analysis for each emotion.

1. *Fear prosody*. In a repeated measures mixed model ANOVA, we confirmed a linear trend, whereby sentences spoken with fear prosody were rated as following: anger scale > sadness scale > happiness scale, *F*(1,103) = 43.86, *p* < 0.001, *η*_*p*_^*2*^ = 0.30. This linear trend did not interact with group membership, *F*(1,103) = 6.66, *p* = 0.42.
2. *Sadness prosody*. Here again a linear trend of fear scale > anger scale > happiness scale (following the evidence suggested by Fig. 2, panel B) was confirmed across both groups, *F*(1,103) = 256.7, *p* < 0.001, *η*_*p*_^*2*^ = 0.71, but did not interact with group membership, *F*(1,103) = 1.9, *p* = 0.17.
3. *Anger prosody*. Anger prosody was not considered as representing any other emotion. This was confirmed by the fact that for both groups, ratings of the anger prosody on the fear, happiness, and sadness scales were lower than 2/6, *t*(79) > 5.58, *p* < 0.001 and *t*(39) > 3.0, *p* < 0.005, for Israelis and Germans respectively.
4. *Happiness prosody*. Similar to anger, happiness prosody was not considered as representing any other emotion for both groups (even if not to the same extent). Ratings of prosodic happiness sentences were lower than 2/6 for Israelis and 3/6 for Germans on all of the other emotional scales (anger, fear, and sadness), *t*(79) > 14.0, *p* < 0.001 and *t*(38) > 2.9, *p* = 0.007 for Israelis and Germans, respectively.

## Experiment 2

As described earlier, one of the main advantages of the T-RES is that it has been adapted to both Hebrew and German, allowing comparisons between the two languages and their speakers using parallel tools. To fully explore similarities and differences between emotional prosodic cues in the two languages, we compared the performance of Israeli native speakers of Hebrew with German native speakers of German using the German version of the T-RES (in preparation).

### Participants

To determine the number of participants in Experiment 2, an a-priori power analysis in G*power (Faul et al., 2009) for a 4 (repeated measures, within) X 2 (between) mixed-model ANOVA for detection of a small effect size (f =.20, a conservative estimate) and a medium correlation between repeated measures (.50) suggested a minimum of 46 participants to obtain .90 power. Anticipating attrition, we aimed to recruit 30 participants per group. Due to COVID-19 outbreak, recruitment was terminated earlier than expected, however the minimum number of participants was obtained (for similar analyses, see Keisari et al., 2022; Nagar et al., 2022). Two groups of participants were recruited for Experiment 2: Israelis—*N* = 24; 15 females, mean age 24.4 years (SD = 0.5) and Germans—*N* = 30; 18 females; mean age 24.4 years (SD = 3.4). The two groups did not significantly differ in age, *t*(52) = 0.08, *p* = 0.93. As gender distribution was not equated across groups, *χ*^*2*^(1) = 0.04, *p* = 0.85, we used gender as a between-group factor in all analyses. Since German is well recognized around the world, we assumed that all Israeli participants would recognize it. However, we made sure that none of the Israeli participants spoke or understood German, as assessed by a self-report questionnaire. All inclusion criteria were the same as in Experiment 1.

## Results

### Israelis identification of German emotional prosody

The first analysis focused only on the Israeli participants, whose mean ratings on the T-RES are shown on the left half of Table 2. We tested whether the Israeli participants could identify the emotions conveyed in the German sentences in the T-RES based only on prosodic cues. Similar to Experiment 1, we conducted a mixed model ANOVA with rated emotion (4: anger, fear, happiness, sadness) and prosodic emotion identification (2: Rated emotion present vs. absent) as within-participants variables, and block order (4) and gender (2) as between-participants variables. We found a large prosodic emotion identification effect, *F*(1, 23) = 493.28, *p* < 0.001, *η*_*p*_^*2*^ = 0.96. Gender and block order did not have a significant effect or interaction with the main factors and will not be further discussed.

**Table 2:** Average ratings on a 6-point Likert scale for the Israeli and German groups with mean emotional ratings of prosodic categories in the T-RES German sentences

Israeli group (*N* = 24)					German group (*N* = 30)				
Rating scale					Rating scale				
Prosody	Anger	Fear	Sad	Happy	Prosody	Anger	Fear	Sad	Happy
Anger	5.6	1.7	1.7	1.5	Anger	5.3	1.5	1.4	1.2
Fear	1.7	5.1	3.7	2.2	Fear	1.5	4.4	3.1	1.6
Sad	1.5	3.8	5.7	1.3	Sad	1.4	2.4	5.4	1.2
Happy	2.0	1.8	1.6	4.4	Happy	1.5	1.3	1.3	5.0

In a follow-up analysis, we tested whether the semantic content of the sentences had an effect on prosodic ratings, by comparing baseline sentences that carried neutral semantic content with sentences that carry emotional semantics. Results indicated that prosodic ratings of sentences with neutral and emotional semantic content did not differ, *F*(1, 23) = 3.0, *p* = 0.092, consistent with our assumption that Israeli participants had no access to the semantic content of the sentences and hence their ratings were based only on the prosody. Therefore, we averaged across semantics in all following analyses.

### Comparison of German native speakers and Hebrew native speakers

**Fig. 3:**
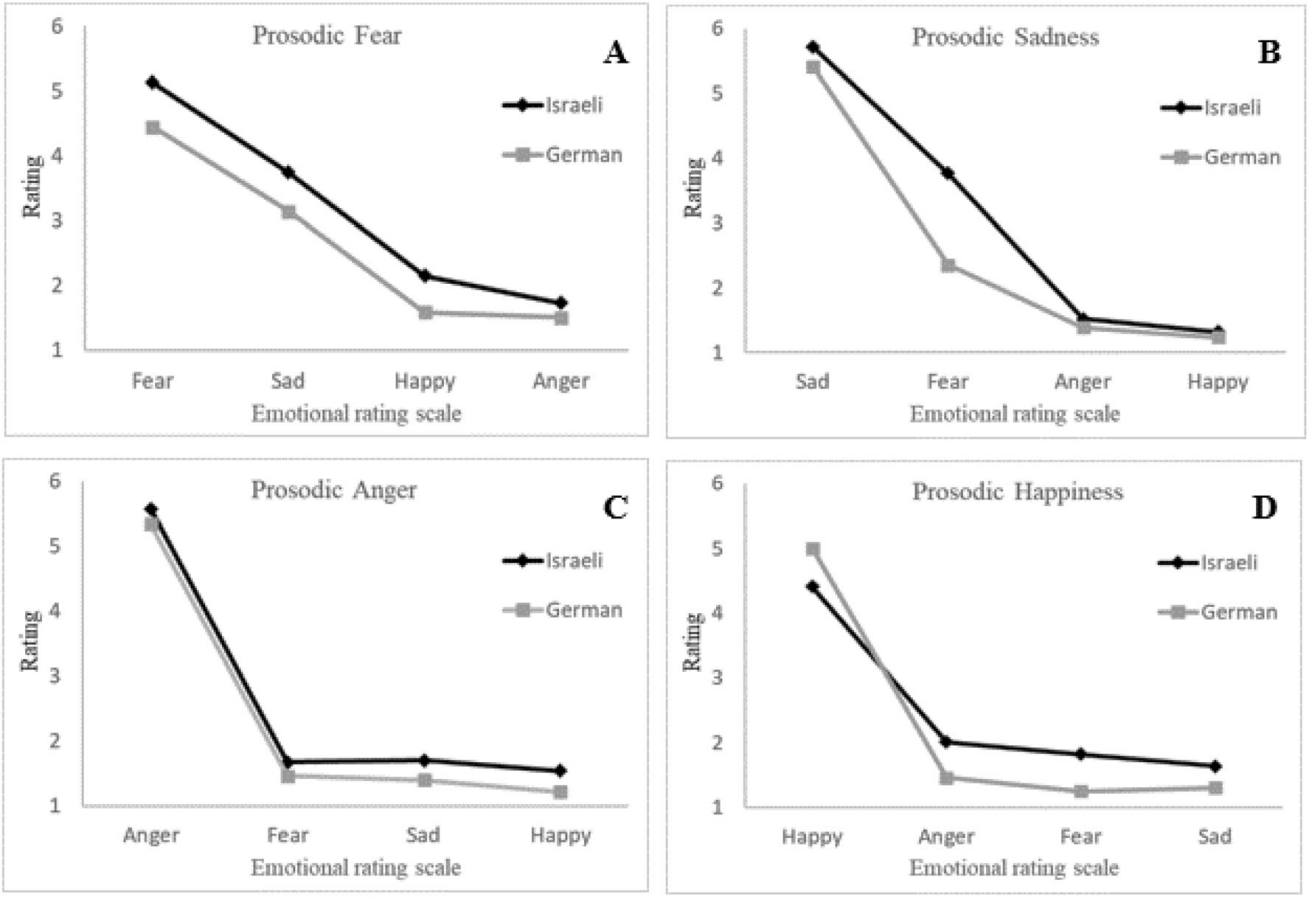
Average ratings of prosodic emotions in the T-RES German sentences. Emotional ratings scale for fear, sadness, anger and happiness

Next, we compared the ratings of the Hebrew native speakers with the ratings of German native speakers, for the same sentences taken from the German T-RES. Table 2 and Figure 3 present the mean ratings on the four emotional rating scales of the respective emotional prosodic categories in the T-RES, for the two groups. We conducted a mixed model repeated measures ANOVA with group membership (2: Germans vs. Israelis), block order (4) and gender (2) as between-participants variable, and with rated emotion (4: anger, happiness, fear, sadness) and prosodic emotion identification (2: Rated emotion present vs. absent) as within-participants variables. First, we found a large main effect for prosodic emotion identification. *F*(1,52) = 1863.73, *p* < 0.001, *η*_*p*_^*2*^ = 0.97, that significantly interacted with group membership, *F*(1,52) = 27.21, *p* < 0.001, *η*_*p*_^*2*^ = 0.35. Planned comparisons revealed that both groups were highly accurate in identifying the prosody, *F*(1,52) = 1320.0, *p* < 0.001, *η*_*p*_^*2*^ = 0.96, and *F*(1,52) = 646.37, *p* < 0.001, *η*_*p*_^*2*^ = 0.93, for Germans and Israelis, respectively, but the extent of the effect was slightly larger for Germans. The difference between the mean rating for the Rated emotion present sentences and the mean rating for the Rated emotion absent sentences was 4.1 for the German group and 3.2 for the Israeli group.

### Identification of discrete emotions

As the last step of analysis, we looked at the average ratings of each prosodic emotion separately. The data as displayed in Figure 3 highlight the many similarities in nuances of the ratings. Two main features can be derived, similar to those in Experiment 1. First, for both groups, on all four prosodies, the highest scores were given on the respective target-emotion rating. In a set of t-tests conducted separately for each group and emotion this was confirmed, for Israelis, *t*(23) > 5.5, *p* < 0.001, and for Germans *t*(29) > 6.6, *p* < 0.001.

Second and more interesting, for both groups, the rating trends on the non-respective emotions were similar as well.

1. *Fear prosody*. In a repeated measures mixed model ANOVA with rated emotion (3: anger, sadness, happiness) as within-participants variable and group membership (2: Germans vs. Israelis) as between-participants variable, we confirmed a linear trend whereby sentences spoken with fear prosody were rated as following: sadness scale > happiness scale > anger scale, *F*(1,52) = 167.16, *p* < 0.001, *η*_*p*_^*2*^ = 0.76. This linear trend did not interact with group membership (*F*(1, 52) = 1.77, *p* = 0.19).
2. *Sadness prosody*. Similarly, sadness prosody was not considered to represent anger or happiness, with ratings lower than 2.0/6, *t*(29) > 8.75, *p* < 0.001 and *t*(23) > 3.1, *p* < 0.005, for Germans and Israelis respectively. Sadness was taken to represent a modicum of fear, as fear ratings of the sadness prosody were the second highest of the four emotional scales, for both Germans and Israelis, *t*(29) > 5.10, *p* < 0.001 and *t*(23) > 9.8, *p* < 0.001, respectively.
3. *Anger prosody*. Anger prosody was not considered as representing any other emotion. This was confirmed, as ratings of the anger prosody on the fear, happiness, and sadness scales were lower than 2.5/6, *t*(29) > 11.4, *p* < 0.001 and *t*(23) > 4.25, *p* < 0.001, for Germans and Israelis respectively.
4. *Happiness prosody*. Similar finding were noted, as the happiness prosody was not considered as representing any other emotion, confirmed by ratings lower than 2.0/6 on the anger, fear, and sadness scales, *t*(29) > 19.85, *p* < 0.001 and *t*(23) > 3.55, *p* < 0.003, for Germans and Israelis respectively.

## Discussion

Prosody is a crucial cue for perceiving the emotional content of speech. It has also been found to be a primary source for the perception of emotion in several languages (Ben-David et al., 2016, 2019). In the present study, we asked whether the prosodic cues used in the perception of discrete emotions are universal or language-specific. Specifically, we sought to determine whether participants with no knowledge of a language could identify the prosody associated with particular emotions in that language, and whether they could do so in a similar way to native speakers of the language in question. To that end, in Experiment 1 we asked both German and Hebrew native speakers to assess the emotions conveyed by the prosody of sentences spoken in Hebrew, which conveyed either anger, fear, happiness, sadness, or no particular emotion. In Experiment 2 we conducted the complementary, asking both groups to assess the emotion conveyed by the prosody of sentences spoken in German. Crucially, the two groups had no knowledge of each other’s language, and thus could not rely on semantic cues to interpret emotional content.

Our first aim was to determine whether listeners could use prosody to identify emotions in sentences spoken in a foreign language, given that no other cues were available. We found that they were indeed able to do so. Both German and Hebrew speakers reliably identified all of the emotions based on prosody alone. Further, they reliably identified which emotions were not present. For example, for a sentence with angry prosody, listeners provided very high ratings on questions about whether the sentence conveyed anger, and very low ratings on questions about whether the sentence conveyed fear, happiness, or sadness. This finding supports those of other studies in the literature based on other types of stimuli, tasks, and language pairs.

Our second aim was to determine the similarities and differences in the use of prosody for identifying emotions in speech between native speakers and non-speakers of a given language. Specifically, we compared the performance of the Hebrew native speakers with the performance of German native speakers on the Hebrew sentences in Experiment 1, and we compared the performance of the two groups on the German sentences in Experiment 2. We found remarkable similarities in ratings across the two groups, despite the fact that the non-speakers of each language were not familiar with the prosody used in that language since they had no knowledge of it. Both groups reliably identified which emotion was conveyed by a given prosody and which emotions were not conveyed by that prosody. As expected, there was a native language advantage in that the Israelis were better at identifying the emotion conveyed by the Hebrew sentences than were the Germans, and vice versa. However, this effect was relatively small. These results are consistent with previous findings in the literature and add information from a new language pair that is typologically very different.

Importantly, using a parallel tool in Hebrew and German allowed us to conduct carefully controlled comparisons unbiased by differences in tool characteristics between the two languages. This comparison yielded similar results for both languages. Specifically, there was no advantage for one group over the other in identifying the other group’s native language prosody. This further highlights the universality of emotional prosody.

The rating-scale paradigm used in the T-RES also allowed us to see more subtle nuances of the identification of emotional prosodies than does the forced-choice paradigm that is typically used in other studies. Importantly, the nuances in the ratings of the German group were very similar to those of the Israeli group in both experiments. Across all emotions, not only were the present emotion sentences rated the highest, but also the order of the ratings of the other three emotions was very similar between the groups. For example, when asked to rate the emotions conveyed in sentences with sad prosody, participants in both groups and both languages rated the sentences in the same order: highest for sadness, then fear, then anger and happiness. Notable similarities are also evident in the relative strength of the ratings across the four emotions. Both groups across both languages assigned the highest ratings to anger and sadness, while both groups assigned the lowest ratings to fear. In addition, both groups had the most trouble ruling out other emotions for sentences spoken with fear prosody. This latter result is consistent with other literature (Ben-David et al., 2013; Pell et al., 2009) showing that fear is one of the most difficult emotions to identify across languages. Additionally, both groups in both languages perceived a considerable amount of fear in sentences with sadness prosody. It is remarkable that the non-speakers mirrored even these very subtle nuances for perception of emotional prosody compared to the native speakers. However, there was a notable difference between the two languages: while in the Hebrew version of the T-RES both groups rated sentences with fear prosody as more angry than sad, when rating the German sentences it was the other way around (here anger was rated even lower than happiness by the Israelis).

The previous paragraph highlighted the overwhelming similarity of perception of emotions with negative valence across the two groups and two languages. Interestingly, the perception of the one emotion with positive valence—happiness—evidenced somewhat larger differences across the groups. The German group rated the Hebrew sentences with happy prosody higher for perception of anger, fear, and sad prosody than did the Israeli group. This is supported by the acoustic analysis of the T-RES sentences, demonstrating larger differences between Hebrew and German for the happy prosody compared to the other emotional prosodies. Specifically, in German happy prosody speech rate was the fastest and significantly faster than in Hebrew (Carl et al., 2022). As an alternative explanation, we note that for the Israelis in Hebrew, the positive happy prosody was accompanied by opposite valence negative lexical semantics (angry, sad and fear). It is possible that the attempts to inhibit the valence-incongruent lexical semantics lead Hebrew speakers to compensate by decreasing rating on opposing prosodic scales. If this were the case, a similar difference should be found for the German sentences. However, ratings of anger, fear, and sad for German sentences with happy prosody were more similar and very low for both groups. Further research is necessary to test whether these results are specific to happiness and to Hebrew, or whether they also extend to other emotions with positive valence, and to other languages.

## Limitations and further research

Our study suggests several interesting directions for further research. Mainly, our results can be explained from both linguistic and cultural perspectives. Future studies should be conducted to test for the relative effects that each of these two important factors has on emotional speech perception and on speech tests in general (see Chu et al., 2021; Icht & Ben-David, 2014). The focus of this study was identification of emotional prosody by individuals who do not speak the language at all. However, it will be interesting to test how this ability will change as function of different levels of proficiency and usage of the language (Chu et al., 2021). Finally, although Hebrew and German belong to different language families (Semitic and Germanic, respectively) they are both non-tonal languages. In these groups of languages, changes in the tone of voice will not change the meaning of the word unlike tonal languages (e.g., Mandarin) where the prosody carries information on the meaning of the word. It will be interesting to test if main trends of our results will be replicated when comparing speakers of tonal and nontonal languages.

## Conclusions and practical applications

In conclusion, the present study provides an important contribution to our understanding of the universality of emotional prosody. It highlights similarities at subtle levels of nuance, even across languages with different typological characteristics such as Hebrew and German. The results of our study have also practical applications. In the global world of the twenty-first century, communication between native speakers of different languages is ubiquitous in daily life. The ability to correctly identify and interpret the emotional message of interlocutors is essential. With the present study, we have shown that even with no access to semantic content, this can easily be done, at least in our German-Hebrew interaction. This knowledge can be of value in designing programs aiming to facilitate cross-linguistic and cross-cultural interactions. In the last decade the world has witnessed large waves of migration as a result of wars and economic crises. Knowledge about the nuances in differences and similarities in emotional prosody can be utilized in language courses for immigrants or in training programs for workers and volunteers of organizations and government agencies that work with this population.

## Supplementary Information

Below is the link to the electronic supplementary material.

Supplementary file1 (DOCX 17 KB)

Supplementary file2 (DOCX 17 KB)
