# Supplementary material for: Processing emotional prosody in a foreign language: the case of German and Hebrew
Source: J Cult Cogn Sci. 2022 Aug 18;6(3):251–68. doi: 10.1007/s41809-022-00107-x (PMC9386669; doi:10.1007/s41809-022-00107-x)
Supplement: Supplementary file 1 — Supplementary file1 (DOCX 17 KB) [file 41809_2022_107_MOESM1_ESM.docx]

| **Prosody** | | | | | |  |
| --- | --- | --- | --- | --- | --- | --- |
| **Neutral** | **Happy** | **Sad** | **Fear** | **Anger** |  | **SEMANTICS** |
| **A6**: Do not waste my time  A7: You brought mud in the house | **A1**: Don't interrupt me  A9: You are driving me crazy | **A13**: You cut me up at the traffic light  **A4**: I am fed up with you being late | **A5**: Enough already with your mess  :**A3** You are crossing every limit | **A8**: You are distracting me  **A2**: The cheek, he overtook me | **Anger** |  |
| **F1**: There is a suspicious object here  **F4**: We need to enter the shelters | **F10**: I hear footsteps in the dark  **F5**: The fire has spread to ours | **F13**: That man is scaring me  **F2**: Someone is following me | **F9**: He has a knife in his pocket  **F6**: I hear shots | **F3**: It is about to explode  **F12**: The dogs attacked me | **Fear** |  |
| **S6**: My friend left the country  **S13**: I feel bad, I feel like dying | **S2**: My dog was run over yesterday  **S3**: I am so alone | **S5**: My aunt died today  **S7**: I am so depressed | **S11**: my life are grey and gloomy  **S12**: My heart is broken | **S1**: I am going to a funeral  **S4**: I cried all day | **Sad** |  |
| **H1**: My team won yesterday  **H5**: I won the lottery | **H13**: Our son was born today  **H2**: I got a pay rise | **H9**: I got the job  **H11**: We really enjoyed the trip | **H8**: Everybody loves me  **H3**: I feel great today | **H4**: I got first place  **H7**: I got a great gift | **Happy** |  |
| NONE | **N5**: The lid of the pot is blue  **N2**: There are hangers in the closet | **N8**: Yotam is wearing a big coat  **N4**: The planet earth is round | **N6**: The monkey climbs on the tree  **N7**: The plate is on the table | **N1**: There are many tables in the room  **N9**: Turn left in the next road | **Neutral** |  |

| **Prosody** | | | | | |  |
| --- | --- | --- | --- | --- | --- | --- |
| **Neutral** | **Happy** | **Sad** | **Fear** | **Anger** |  | **SEMANTICS** |
| **A6** אל תבזבז לי את הזמן  **A7** הכנסת בוץ הביתה | **A1** אל תתפרץ לי לדברים  **A9** אתה מוציא אותי מדעתי | **A13** חתכת אותי ברמזור  **A4**נמאס מהאיחורים שלך | **A5** די כבר עם הבלגאן שלכם  **A3** אתה עובר כל גבול | **A8** אתה מפריע לי להתרכז  **A2** החצוף הזה עקף אותי | **Anger** |  |
| **F1** יש כאן חפץ חשוד  **F4** צריך להכנס למקלטים | **F10** אני שומע צעדים בחושך  **F5 האש מתפשטת לעברנו** | **F13** האיש הזה מפחיד אותי  **F2** מישהו עוקב אחרי | **F9** יש לו סכין בכיס  **F6** אני שומע יריות | **F3 זה עומד להתפוצץ**  **F12**הכלבים תוקפים אותי | **Fear** |  |
| **S6** חבר שלי עזב את הארץ  **S1 רע לי בא לי למות** | **S2 הכלב שלי נדרס אתמול**  **S3** אני כל כך בודדה | **S5** דודה שלי נפטרה היום  **S7** אני כל כך מדוכאת | **S11** חיי אפורים וקודרים  **S12** הלב שלי נשבר | **S1** אני הולכת ללוויה  **S4**בכיתי כל היום | **Sad** |  |
| **H1** הקבוצה שלי ניצחה היום  **H5** זכיתי בלוטו | **H13** נולד לנו בן היום  **H2** קיבלתי העלאה במשכורת | **H9** התקבלתי לעבודה  **H11**נהנינו מאד בטיול | **H8** כולם אוהבים אותי  **H3** אני מרגישה נפלא היום | **H4** הגעתי למקום הראשון  **H7** קיבלתי מתנה נהדרת | **Happy** |  |
| NONE | **N5** המכסה של הסיר כחול  **N2** בארון יש קולבים | **N8** יותם לובש מעיל גדול  **N4** כדור הארץ הוא עגול | **N6** הקוף טיפס על העץ  **N7 הצלחת על השולחן** | **N1** יש בחדר הרבה שולחנות  **N9** ברחוב הבא פנה שמאלה | **Neutral** |  |
