# Supplementary material for: Processing emotional prosody in a foreign language: the case of German and Hebrew
Source: J Cult Cogn Sci. 2022 Aug 18;6(3):251–68. doi: 10.1007/s41809-022-00107-x (PMC9386669; doi:10.1007/s41809-022-00107-x)
Supplement: Supplementary file 2 — Supplementary file2 (DOCX 17 KB) [file 41809_2022_107_MOESM2_ESM.docx]

**Examples for German sentences for four emotional categories and neutral**

| (Emotional) Category | Sentences |
| --- | --- |
| Anger | Du gehst mir total auf die Nerven |
|  | *You are a pain in the neck* |
|  | Provoziere mich bloß nicht noch weiter |
|  | *Do not provoke me any further* |
|  | Kümmere dich um deinen eigenen Mist |
|  | *Take care of your own crap* |
|  |  |
| Fear | Ich verlier die Gewalt über mein Auto |
|  | *I’m losing control over my car* |
|  | Das Feuer hat die Gasleitung fast erreicht |
|  | *The fire has almost reached the gas line* |
|  | Es kann jeden Moment explodieren |
|  | *It can explode any moment* |
|  |  |
| Happiness | Meine Mannschaft hat gestern gewonnen |
|  | *My team won yesterday* |
|  | Ich fühle mich heute großartig |
|  | *I feel great today* |
|  | Seine Worte bringen mich zum Lächeln |
|  | *His words make me smile* |
|  |  |
| Sadness | Mein Hund musste eingeschläfert werden |
|  | *My dog had to be euthanized* |
|  | Du fehlst mir an jedem einzelnen Tag |
|  | *I miss you every single day* |
|  | Niemand interessiert sich für mein Leben |
|  | *Nobody is interested in my life* |
|  |  |
| Neutral | Der Kunde kauft eine graue Hose |
|  | *The customer buys a pair of gray trousers* |
|  | Der Teller steht auf dem runden Tisch |
|  | *The plate is on the round table* |
|  | Meine Tasche liegt im Zimmer |
|  | *My bag is in the room* |
